# Surrogate labour: exceptional for whom?

**DOI:** 10.1080/03085147.2018.1487180

**Published:** 2018-07-25

**Authors:** Bronwyn Parry

**Affiliations:** School of Global Affairs, King’s College London, Room 4.19 Bush House North East, 40 Aldwych, London, United Kingdom

**Keywords:** assisted reproduction, surrogacy, clinical labour, exploitation, surrogate mother, India

## Abstract

The desire for genetically related children is driving an exponential rise in assisted reproductive service provision worldwide, including the Global South. In India, the number of ART (Assisted Reproductive Technology) clinics has more than doubled over the past three years. This expansion has been accompanied by a similarly explosive growth in populist narratives that assert that one of the services offered by such clinics, commercial gestational surrogacy (CGS), is a form of labour that is so exceptional(ly) exploitative it should be banned. Provocative headlines proclaiming that surrogates are ‘Renting their wombs’ and ‘Pimping their pregnancies’ fuel such assertions, suggesting that surrogates become reduced to mere wombs, vessels for carrying the offspring of entitled and wealthy foreigners. Although superficially compelling, such arguments fail to withstand detailed interrogation. Utilizing insights from anthropology, the history of science and law and bringing to bear the findings of extended fieldwork in Mumbai, Jaipur and Delhi, I critically analyse three assertions made in relation to surrogacy: that it is (i) a particularly intimate or invasive form of bodily exploitation; (ii) a uniquely sacralized form of affective labour and (iii) a uniquely generative form of labour. In arguing against exceptionalism, I contend that such practices cannot be adjudged through application of universal ethical principles and norms, but rather must take account of the complexity of the lived experience of all the participants, placed in their sociological and geographical contexts.

## Introduction

A distinct advantage of approaching research on new human reproductive technologies via the history of animal and plant modification is the ability it affords to recognize that for every apparently ‘unprecedented’ development in the engineering of biological life there is, if not a precedent, then at least a significant precursor. This is certainly true of the practice of assisted reproduction. Popularly imagined to have begun with the birth of Louise Brown, the world's first ‘test tube’ baby in 1978, artificial insemination had, in fact, first been perfected by the Italian natural philosopher Lazarro Spallanzani nearly 200 years earlier in 1779. Unconvinced by theories that life was generated through preformation or the action of a ‘vegetative force’ (something akin to the Aristotelian notion of an *aura seminalis*), Spallanzani set about tracing the role of what he described as ‘spermatic worms’, this ‘race of little animals’, to the end. Designing ingenious methods to collect the seminal fluid of excited frogs (including clothing many dozens of them in waxed taffeta britches), he established not only that the ‘liquor’ was essential to conception but that he could himself intervene in this process, serving as the very instrument of the intermingling. This practice of ‘artificial fecundation’, as he called it, succeeded, miraculously, ‘as well as if the male himself had performed his proper function’ (Pinto-Correia, 1997, p. 197).

Allied techniques including the freezing of bodily fluids and tissue had begun even earlier with Francis Bacon's first attempts to preserve chickens by stuffing them with snow, experiments that unfortunately precipitated his untimely death from pneumonia. His contemporary Robert Boyle's further investigations into the effects of super-cooling on organisms were advanced significantly in the late nineteenth century with the liquefaction of the permanent gases. Their application in the realm of human reproduction culminated in the mid-1950s in the successful freezing and thawing of vitrified human eggs and sperm and the establishment of the discipline of gamete cryobiology. Situating contemporary developments in assisted reproduction within this long durée of technological change is important for at least two reasons: it makes it possible to establish when significant ontological or epistemological ruptures between past and present practices occur; and to determine *how, if, and in what ways* they act to alter our understanding of, or relations to, the social world. In short, it enables us to ascertain when things become genuinely ‘exceptional’.

On this reading, it is possible to conclude that these technological developments did institute a genuinely unprecedented moment in the history of science. Until this time, the longest any gametes had retained their generative capacity outside of the body was some frozen rabbit semen that had, in 1926, survived for 12 hours on the overnight train from Cambridge to Edinburgh. The introduction of advanced cryogenic technologies was, however, set to radically transform the existing dynamics and organization of cellular life. With their introduction, it became possible for tissues, including gametes, to be extracted and disassociated from the bodies in which they were produced and to be stored for more than 100 years without any loss of vitality.

The spatial and temporal parameters of their ‘natural’ existence were thus able to be extended in ways that surpassed any previous imagining. This was significant as gametes became, for the first time, capable of entering a state of *immortalization and hypermobility*: able to be kept in a viable state of suspended animation; and capable of being circulated, firstly, into the artificial body of the gamete bank, then on to laboratories where they could be fertilized to create embryos, before being distributed to their waiting recipients. These could include the women from whom they were originally sourced (following a temporal lag), but also others located across the globe, from California to Cambodia, who had agreed to have such embryos implanted in them and to carry them to term, either altruistically or for payment.

As Marilyn Strathern (1992, 2011), Sarah Franklin (1995, 2013) and Charis Thompson (2005) have argued, assisted reproductive techniques disrupt conventional ideologies that have historically essentialized and valorized the genetic connection between mother and child. Motherhood undergoes a necessary reconceptualization as it is disaggregated into its new registers: genetic, birth, adoptive and surrogate. These ‘multiple maternities’ as Inhorn and Birenbaum-Carmeli (2008) call them, split apart, both socially and practically, the previously unified work of maternal labour, ushering in what Strathern (1992, p. 27) describes as ‘a new convention: the distinction between social and biological parenting’. Add to this the concept that some women could be *paid* to carry another's child, allowing maternal labour (conceived of by many as the most sacred of acts) to become commodified, and we have, it would seem, all the conditions necessary to spark a storm of social condemnation. The backlash against commercial surrogacy, particularly that provided by women in developing countries, has been both intense and sustained, amplified considerably in recent years by a deluge of press that characterizes the provision of such services as highly exploitative. Provocative headlines proclaiming that surrogates are ‘Renting their wombs’ (Sinha, 2011) and ‘Pimping their pregnancies’ (Bindel, 2016) generate a compelling and increasingly familiar narrative: that such women are being coerced or deceived into a trade in which they are reduced to mere wombs, vessels for carrying the offspring of entitled and wealthy foreigners.

The alarmist tenor of such accounts has also travelled, circulating beyond the blogosphere to inflect the discourse of those operating at even the highest levels of government and the judiciary today. In October 2015 two justices of the Indian Supreme Court, Justices Ranjan Gogoi and N. V. Ramana, called for a total ban on the provision of commercial surrogacy to all foreign nationals, pronouncing that ‘renting a womb … amounts to the economic and psychological exploitation of the surrogate mother and is inconsistent with the dignity of womanhood’.^1^ To near-universal astonishment, and despite the fact that a revised Bill regulating assisted reproduction was then in its final stages of negotiation, the Indian government agreed to introduce just such a ban. What began as legal opinion has since been formalized into *The Surrogacy (Regulation) Bill* 2016, an Act that criminalizes all forms of commercial surrogacy, even those commissioned by Indian nationals. That surrogacy as an activity was sectioned out of India's wider *Assisted Reproductive Technology (Regulation) Bill* would appear to sustain the implicit assertion that commercial surrogacy constitutes a uniquely exploitative form of labour that demands singular attention and bespoke forms of regulatory address.

My intention in this paper is to take this powerful assertion and to scrutinize it forensically. I do so, not because I think that commercial surrogacy cannot be exploitative, but rather because I am unconvinced by arguments that it is inherently or necessarily so.^2^ Appeals to exceptionality must, if they are to be sustained, survive crisp theoretical and empirical analysis. Drawing on four years of intensive empirical work within India's burgeoning Assisted Reproductive Technology (ART) sector,^3^ I consider how, and in what ways, assertions about the fundamental immorality of commercial gestational surrogacy (that emanate primarily from Western philosophical traditions) sustain when translated into practice in divergent localities and sociological contexts. The contention that commercial surrogacy is an exceptionally exploitative form of labour appears to rest on two sets of arguments. The first is that there is something distinct about reproductive labour itself that justifies its exclusion from the realm of commodification, that it to say, that makes its commercialization abhorrent; the second, that it is something about the social or spatial organization of that labour that makes it exceptionally exploitative and thus requisite of specific legislation. It is my intention to subject both claims to detailed interrogation here.

## Why fetishize reproductive labour as exceptional?

A range of recent literature has carefully explicated the ways in which the generative capacities of ‘life itself’, to employ Nikolas Rose's term (Rose, 2009), have recently been drawn into regimes of commodification. ‘Life itself’ here includes not only alienated tissues, organs, reproductive cells and DNA, but also forms of embodied labour that have become key generators of value in the bioeconomy. Sociologists Melinda Cooper and Catherine Waldby argue that the *in vivo* biology of human subjects, that is to say an individual's very corporeality and its capacity to endure, to sustain or to generate life, is becoming an increasingly valuable resource to the life sciences industries. As they astutely note, these industries have come to rely ‘on an extensive, yet unacknowledged labour force, whose service consists in the visceral experience of experimental drug consumption … ejaculation, tissue extraction and gestation’, forms of work that they go on to characterize as ‘clinical labour’ (Cooper & Waldby, 2014, p. 7).

They argue, following Boltanski and Chiapello (2005), that post-Fordist models of economic organization and governance have invoked profound shifts in the organization of labour. We have moved, in the West at least, from an economy that provided near full employment and strong forms of statutory labour protection for industrial workers to a system that favours outsourcing to private contractors. This, in turn, results in more precarious conditions of work and greater exposure to occupational health and safety risks. Clinical labourers, they suggest, are particularly vulnerable: exploited financially, in unstable outsourced employment, working under oppressive contractual relationships, they have become the contingent workers of the bioeconomy, the victims of a voracious neo-liberalism. Gendered and racialized divisions of labour are said to further amplify these inequities (Thompson & Sofio, 2014). Oppressed minorities and women of colour who cling on to life at the margins of human existence are argued to be especially susceptible to exploitation, with women's reproductive capacities becoming the target of a wider marketization of biological vitality. Earlier sociological studies (Baylis & McLeod, 2007; Kirby, 2014; Macklin, 1988; Nelson, 2013; Van Niekerk & Van Zyl, 1995) have characterized both gestational surrogacy and egg vending as malign practices in which women can be pressured to provide contractual use of their bodily capacities to others for payment in ways that compromise both their autonomy and their dignity.

Whilst such analyses are extremely useful, they do raise the question of why these particular forms of reproductive labour should be fetishized as exceptional (and thus prohibited from commercialization) or *how* indeed they differ from earlier historical modes of contractual bodily exploitation. The argument seems to rest on three popular assertions: that reproductive labour is (i) a particularly intimate or invasive form of bodily exploitation; (ii) a uniquely sacralized form of affective labour; or (iii), a uniquely generative form of labour. These factors together, it is argued, serve to set reproductive labour apart from the other forms of labour in which we routinely engage, and for which we are unproblematically recompensed. It is to an unpacking of these core assumptions that I first wish to turn.

It is certainly true that carrying a child for another is a particularly intimate form of bodily labour, but is it any more so than some of its historical antecedents? At all stages of human development and in all economic systems from the feudal to agrarian, industrial to post-industrial, the unique corporealities of particular individuals, from the hulking strength of the stone mason to the wiry resilience of the rickshaw pullers, have been exploited for profit often under precarious forms of contract labour. Historically, the argument that ‘bodies themselves’ are unimportant in wage labour exchange as individuals are, in effect, selling their abstracted, alienable labour rather than their embodied selves has, as O’Connell Davidson (2014, p. 516) astutely notes, (citing Davidoff) only served ‘to reproduce “the fiction of disembodied actors with the capacity to sell labour away from the person (the body) of the labourer”’. In fact, sacrificing the fabric of one's very being in the service of paid employment, whatever that employment might be, should, in my view, always be understood as an intensely intimate form of bodily labour. It remains a curiosity that the term ‘bodily labour’ seems reserved solely for acts that involve paid use of bodily parts designed for procreation or reproduction.

Abolitionists routinely conflate gestational surrogacy with prostitution (which they confusingly argue is also a *unique* form of self-commodification) in their attempt to draw parallels between what they consider to be two equally pernicious expressions of female subjugation under conditions of patriarchal domination. In so doing they extol the narrative that, while labour power is generally understood in the liberal tradition as a form of property that is separable from the person, and thus able to be justly alienated for sale to the highest bidder, sale or rent of one's intimate sexualized self cannot be similarly or unproblematically abstracted away from the essence of the seller's personhood and must consequently remain outwith the realm of commodification. What such conceptions obscure, however, are the very many commonalities that such labour shares with other allied forms of reproductive, affective and emotional labour. These, I suggest, work to collapse, rather than sustain, arguments for exceptionality.

Let us take, by way of example, two other forms of highly intimate bodily labour that have long histories of commodification. As Sussman and others have noted (1982, p. 3), wet nursing, ‘the practice of sending newborn children away to be suckled by rural women for pay’, was commonplace in Europe from the middle ages onwards, whilst the practice of donating sperm, although initially unrecompensed, has subsequently become a recognized form of waged labour for men across the globe. Both are clearly intimate forms of bodily labour that have been unproblematically commodified. The exceptionality of gestational surrogacy must therefore rest on some other quality of the expended labour. It is at this point that three further arguments are brought into play: that carrying a child is a uniquely invasive form of bodily labour, that it is a sacralized labour unlike any other and that it is, in some way, uniquely generative.

It has been argued that whilst wet nursing or sperm donation are practices that involve bodily intimacy, this labour is neither invasive nor risky. The resources that are commodified (breast milk and semen) are naturally renewable, and contributing them is, it is suggested, undemanding, if not even potentially pleasurable. While some countervailing evidence suggests that managing either as a form of paid employment is by no means as effortless as may be first presumed (Almeling, 2011), they do not typically involve the *internalization of risk*. Surrogacy and egg vending, conversely, involve the ingestion of drugs such as ovarian hyperstimulants that can induce pain, abdominal inflammation, possible renal failure and even, in 5 per cent of cases, the onset of ovarian hyperstimulation that can result in premature death. This, along with the corporeal insults that might result from carrying a multiple pregnancy must surely provide substantiating grounds for exceptionality?

This argument, in my view, lacks purchase. Many of the alternative forms of employment open to this constituency of workers are, sadly, equally if not more invasive and injurious to health. Large numbers of young women who work for less than two US dollars a day in tanneries on the outskirts of Kolkata and Mumbai are routinely exposed to highly toxic chemicals such as chromium sulphate, formaldehyde, azocolourants and pentachlorophenol that induce terrible skin conditions and respiratory diseases and dramatically increase their rates of cancer. Other mothers and children who survive by recycling batteries and e-waste in their homes suffer similar levels of extreme toxic exposure. In these cases, unlike those associated with assisted reproduction, exposure to harm is rarely limited temporally. These individuals are subject to corporeal insults that accumulate over decades rather than months, inevitably magnifying their health impacts. This is not to disavow the fact that wrongful harms may result from malpractice or a lack of enforcement of appropriate conditions of work for surrogates or egg donors, but rather to draw attention to the fact that whilst these are lamentable, they remain as amenable to remedy (or not) as any inadequate working conditions are, for any person, in any under-regulated sector of the Indian economy.

A second set of arguments suggest that maternal labour (the gestation and birthing of a child and its immediate post-partum care) is a sacred act, a form of profoundly affective labour that is sullied by commodification. Affective labour, the largely invisible yet intense work of producing and managing emotions in and between others (nurturing, comforting, listening, caring, protecting), has historically been understood as a highly feminized and usually unrecompensed labour. However, in a post-Fordist world in which industrial production has been largely displaced by a service economy, the commodification of all manner of care work has, I would argue, become entirely normalized. As Hardt and Negri put it, ‘the production of “common forms of wealth” such as information, affects and social relationships are now routinely expropriated by capital to generate surplus value’ (2011, p. 139).

The work of the economic sociologist Viviana Zelizer is particularly useful here in explicating the complex relationships that exist between money and forms of intimate or affective labour. As she has noted, many commentators and scholars like to foster the notion that intimate relations and economic activity occupy distinct domains, the former ‘a sphere of sentiment and solidarity’, the latter ‘a sphere of calculation and efficiency’ (2005, pp. 20–21). We are invited to imagine that each remains hermetically sealed from the other and that contact between the two will result in ‘moral contamination’. This doctrine particularly animates the concerns that attend the commercialization of reproductive labour and the potential this is thought to have in corrupting the sacredness of the act of child-bearing. The fear that money will debase the sanctity of this uniquely intimate bond is palpable.

And yet, an unflinching inspection of our own social practices reveals that many in the West have no compunction whatever about commodifying all manner of commensurable, intensely intimate forms of labour. We now routinely pay others to perform highly intimate personal care work for our elderly kin with dementia and think nothing of asking nursery workers to provide long-term substitute parenting to children, and indeed babies, of only a few months or even weeks of age. The fact that it proves so difficult to find online images of the most profoundly affective examples of this kind of labour – comforting sobbing children or trying to read the desires of the profoundly disabled – is perhaps a testament to our unwillingness to acknowledge how readily we delegate such intimate work to pay others whilst simultaneously concealing that fact. Any assertion that the affective labour performed by the surrogate in bearing, or caring for, the commissioned child is substantively distinct from these would have to be predicated on an argument that is much more consistently applied than it is now.

What becomes evident is that economic relations are not only present in these realms but are vital actuators of this key relational work. Intimacy and economic transactions are not ‘strange bedfellows’ but rather are brought into productive engagement every day as we construct the means to fulfil our domestic and professional needs. Only once we accept that caring labour has always been commodified can we understand, as Zelizer (2005, p. 307) puts it, ‘that some negotiated matches involve injustice, cruelty, damage, or confusion, *not because they mix economic activity with personal relations*, but because they result from improper exercises of power’ (emphasis added). The question to concentrate on here, as she concludes, is thus: ‘which arrangements for personal care of children, the elderly, the disabled or the sick damage the recipients, the caregivers and the households involved … and which arrangements actually enrich participants’ lives’ (2005, p. 306)? This is a matter to which I shall return shortly.

A further commonly made assertion is that reproductive labour should be considered exceptional because it is uniquely ‘generative’ in nature. This is an argument with which I have more sympathy, yet it too bears closer analysis. Reproductive labour is said to be exceptional as it involves giving life to something unique (the child) that the surrogate is then asked to relinquish. To do so requires a certain partitioning of sentiment from action. It is undoubtedly true that many women would find this impossibly difficult to do. Others, however, can come to an accommodation with relinquishment.^4^ This may seem unlikely or surprising to some, although it remains the case. Some may have less maternal instinct; others, conversely, view the commissioned child as an endowment or legacy that they are content, if not proud, to bequeath to others.

There are clear commonalities and resonances here with the experiences of those who work in the inventive economies of the modern university, computer lab, architectural office or engineering facility. We too also receive remuneration (hopefully, if we put the gender pay gap aside, of an appropriate amount) to employ our corporealities, our bodies and brains, to generate unique ideas, outputs and manufactures. These are also surrendered to others who are encouraged to adopt and develop them on their own terms. Perhaps what all of us (surrogates included) are generating are what my associates in intellectual property law would call ‘creative works’. Books, software or inventions appear inanimate but, as they are released into the world, also come to take on a life of their own, to acquire their own ‘careers’ as the anthropologist Arjun Appadurai (1988) would say. Like surrogates, we retain, as their authors, a keen interest in their respective life courses, knowing, even so, that their trajectories will be ones that even we will have limited powers to shape.

## Surrogacy: exploitative for whom?

If, then, there proves to be more commensurabilities between the work of gestating children and other forms of bodily, affective or generative labour than we first might have imagined, it must then be the conditions under which this reproductive labour is performed that serve to make it so exceptionally exploitative, such an affront to moral dignity. Let us turn now to consider this thesis. The recent ban on the offering of commercial surrogacy services in India today is predicated on the argument that the practice is highly exploitative of the women involved – in fact, we must presume, uniquely exploitative, since it is this practice alone amongst various kinds of clinical labour (including, for example, participation in clinical trials) that has been criminalized and singled out for prohibition. We must then begin by asking the questions: ‘what is exploitation, and exploitative for, or by, whom?’

The agreed dictionary definition of exploitation is: ‘the action or fact of treating someone unfairly in order to benefit from their work’. Almost all of the most vocal interlocutors in the ‘reproductive-labour-in-emerging-economies-is-inherently-exploitative’ debate would fashion themselves as concerned liberals who take the view that the imbalances that exist in the purchasing and earning power of the surrogate or egg donor and the commissioning parents (who are assumed to be wealthy foreigners) invoke a classic dynamic of exploitation in which the economic desperation of the surrogates leads them to downplay or discount risks to their health or psychological well-being that would otherwise deter them from engaging in such practices (Deveaux & Panitch, 2017; Phillips, 2017).

‘Informed consent’, even if secured, is presumed to be invalidated when obtained under conditions of structural violence or injustice. Whilst, in theory, we might concur with these normative principles, in practice, their application becomes much more complex. I believe that it is only by situating these practices within the lived experiences of these women, by sensitively contextualizing their motivations for undertaking such work within their own life worlds, that we can arrive at a more nuanced reading of whether such work is either morally or ethically exploitative. In bringing these women's voices back into the debate we can, as it were, ‘flip the classroom’ by asking of ourselves that vital anthropological question: what work does framing commercial surrogacy or egg donation as exceptionally exploitative perform … and for whom?

So, let us begin with the women: who are they? Why do they undertake this work and, more importantly, for whom? In answering these questions, I draw on four years of fieldwork in Mumbai, Jaipur, Delhi and Bangalore and interviews with 123 individuals working in the assisted reproduction economy there, including many surrogates, sperm and egg donors, clinicians and commissioning parents. So, are these women, as Waldby, Cooper and others have argued, an underclass that exist at the margins, oppressed, exploited financially, in unstable outsourced employment? The answer seems to be consequential on the question of where one locates ‘the core’. These clinical labourers certainly do not enjoy the financial security and economic and social advantages of (some) women in the metropolitan West, and it is certainly true that surrogates and egg donors have historically been drawn from the lower castes and classes of India, although, and this is an important point, not *the* lowest classes. Ironically the very precarity of that cohort's existence – the impermanence and shabbiness of their accommodation and their vulnerability to malnutrition and disease – usually serves to exclude them from such employment.

Surrogates are much more likely to have been recently employed in other related sectors of the service economy in work that involves the provision of closely allied forms of affective or bodily labour such as live-in domestic service, aged or child care work – employments in which many have been subjected to extreme financial or sexual exploitation or coercion. Others labour producing piece work at home or in garment factories, where, as Rudrappa's research reveals, they endure a litany of assaults to their physical and psychological well-being, ranging from respiratory difficulties to urinary tract infections, from hearing loss to the stress induced by 14-hour shifts and sequestration in hostel-style dormitories (2012; Rudrappa & Forest, 2014). Their subjugation to these highly oppressive working conditions results in what we routinely acknowledge (if not accept) to be the *mundane exploitation* of the lower classes. Mundane is a useful term here as its derivation from the Latin *mundanus* or ‘belonging to the world’ rightfully locates such conditions as universally present in all domains of the global capitalist economy. Whilst far from a cause for celebration, neither does this serve to make commercial surrogacy uniquely exploitative nor worthy of highly stylized disapprobation that these other, significantly more damaging, forms of labour rarely attract. For the women we interviewed surrogacy provided a rational and carefully considered means to escape these exceptionally dangerous or onerous employments for what they consider to be a much more secure, economically productive and personally rewarding form of work.

Moral philosophers such as Wertheimer (1999) and Sample (2003) concur that exploitative transactions arise when the compensation or market rate for services offered to providers is so low that it fails to improve their situation sufficiently, whilst the exploiter profits unduly from the exchange. Such assessments are, however, extremely difficult to undertake through appeal to a set of abstract normative principles – context here, again, plays a key role in determining what is, and is not, a just or *sufficient* form of compensation. Our own research has determined that many women find the payment that they receive to undertake surrogacy (£4–5,000 or the equivalent of more than five years of income in their previous occupations) to be more than ‘sufficient’ to transform their own lives and to have considerable intergenerational benefits for family members, particularly their female children. In fact some reported that clinicians had advised them to invest the money they receive and even directly assisted them in purchasing property.

Madam bought this house herself for me. She told me that the money that you get you should not spend it away, buy a house, and live there, and in this way you can save the rent money also. So I now have a nice house, and my children are also happy. My daughter is also doing well and she is educated and acting in films. My elder son is studying and my younger son is also studying. Both are studying well and my life is good. (Bhagwati – surrogate)

Clearly, the purchase of education, housing, healthcare or business infrastructure can have life-enhancing effects, the positive impacts of which cannot be readily disavowed or discounted. Of course the capacity to fully realize such benefits is unevenly distributed. As other studies have shown, some surrogates are less adept at financial management and have not used the payments to effect lasting change in their material circumstances. Nevertheless, it is easy to overlook or downplay the profound and lasting alterations in the personal and political dynamics of their lives that result from receiving a payment of this magnitude. It is, almost without exception, the first time that such women have brought into their households income that substantially outperforms that of their male partners or relatives, having done so, notably, through the performance of a form of labour that those male figures can never emulate. The enhancements to their agency and autonomy (as reflected in the household's acquiescence to their continued work in the field as either surrogate or agent) that can arise as a consequence are transformative, both domestically and culturally. In these terms the affective changes may prove to be more significant than the material ones.

Would the sum that they receive for undertaking a surrogacy be deemed ‘sufficient’ if they were performing the same labour in, say, London? Clearly not, but that would be a consequence of wider structural divergences in both earning power and living costs. Similar discrepancies attend all forms of waged labour performed within, and between, the world's more or less advanced economies. To argue that surrogacy is exploitative simply because surrogates are paid *only* £4,000 in Mumbai, but £15,000 in Newcastle, is both naïve and fallacious. It is, however, pertinent to question whether it is exploitative for the wealthy to take unfair advantage of these structural discrepancies in securing a service (surrogacy) for which they would elsewhere pay considerably more. That argument would certainly sustain *if* an equivalent service could be readily accessed in the purchasers’ immediate locality with increased cost the only deterrent to uptake. This is, strangely, not the case. Decisions by regulatory agencies (such as the UK's Human Fertilisation and Embryology Association, HFEA) to abdicate responsibility for regulating surrogacy in practice have facilitated the emergence, in the United Kingdom, of a precariat of fully unprotected surrogate workers and a flourishing shadow economy rife with deceptive practices. Such developments have, ironically, served to drive many commissioning parents to what they perceive to be the relative safety of more highly regulated clinics located in Mumbai or Delhi's most prestigious hospitals.

This takes us then to a consideration of the terms and conditions of employment of the Indian surrogates who work in, or for, these clinics – the contractualization of their labour and the degree to which their consent can be considered to be truly informed. Here we find another complex set of dynamics in play. Much has been made of the fact that Indian surrogates are largely unaware of the terms and conditions of their employment. Our research suggests that that is true in terms of their awareness of the medical risks and potential for complications, but less so apropos the agreed financial rewards. Payment was a clear motivational factor amongst the surrogates we interviewed, and they, their immediate families and agents were proactive in securing these payments. It was clear, however, that many surrogate women had only limited understanding of what they would be subjected to corporeally and certainly lacked any authority to determine ‘treatment’ pathways. Most are poorly informed about the pharmaceutical regime they will be required to undertake; the risks of ovarian hyperstimulation; the number and destinations of their harvested eggs; the number of embryos that will be implanted in them and the consequent risk that they may be subjected to a foetal reduction.

These are undoubtedly very serious matters, and they could constitute exploitative practice. But are they necessarily or inherently exploitative? I would argue not. The work of framing these practices as such is only achieved by conflating arguments about what is ethically insupportable with that which is morally objectionable. It is clear, for example that these troubling examples of poor care simply constitute forms of medical malpractice that are ethically unacceptable but which remain amenable to remedy through regulation. The fact that they exist in some contexts in India simply reflects the uneven landscape of care that characterizes the provision of assisted reproduction worldwide. We know, for example, that in many other economies and jurisdictions such as the United Kingdom, Canada, Australia and the United States practitioners have established protocols to optimize patient welfare when undergoing procedures such as ovarian stimulation, embryo transfer, foetal reduction, egg donation and the like (Thompson, 2007). Why can these not be instituted as global norms of clinical best practice in the ART sector to level up standards of care and address uneven geographies of regulation? Exploitation here arises largely as a consequence of avertable failings in the regulatory environment rather than being an artefact of something that is inherently morally abject in the practice of surrogacy itself. In other words, it is in the mechanics of the practice, not in the principle itself, that the trouble lies.

## Moral economies of distributed kinship

In addressing this point let us then lastly move from an examination of the social, political and economic circumstances of this form of reproduction to an analysis of the moral economy of surrogacy and egg donation. The argument that commercial surrogacy is morally abject, despite any of the terms and conditions of its practice, hinges, I believe, on two presumptions: (i) that it is simply wrong to bear a child for another for payment, or (ii) that commissioning is driven by economic privilege and expediency. Much has been written about the purported commodification of the child with which I have little patience. Let us consider this bold assertion: that those of us who have them have all ‘paid’ to acquire our children in one way or another, be that through the provision of courtship gifts, meals and entertainments, engagement rings, expensive weddings, salary support and the like. All of these transactions constitute a means of inducing or cross-subsidizing the production of children. A more unabashed approach to paying for such labour might jar, but is it really all that distinct from the commodification of all manner of intimate exchanges, a practice in which we all routinely engage but systematically elide?

Is the commissioning of surrogacy driven by economic privilege and expediency? The assertion here is that reproductive labour is, like other forms of corporeal or affective labour (such as child care or domestic work), something that commissioning parents could probably do for themselves but do not, as they lack either the time or the inclination – displacing that labour inappropriately to others by virtue *only of their ability to pay to do so*. What is termed ‘social surrogacy’ or sometimes ‘vanity’ or ‘designer’ surrogacy is said to arise when women (stereotypically portrayed as highly paid barristers or Bollywood stars) who are capable of bearing their own children expatriate this labour to others in the interests of protecting their career trajectories or bodily integrity. Whilst this has become a popular leitmotif for the tabloid press, we saw no evidence of it in practice during our fieldwork. In all of the cases we saw, commissioning parents were seeking surrogates not because they were too lazy to have a child of their own but because they were experiencing specific forms of untreatable structural infertility (uterine abnormalities, for example). Whilst some celebrities have availed themselves of the use of surrogates, closer analysis reveals that they also often do so as they are infertile, or in the case of homosexuals, unable to reproduce biologically related children without such assistance.

Very interestingly, many of the commissioning parents we interviewed were not foreigners but rather India's rising middle classes for whom surrogacy is becoming increasingly normalized. Moreover, despite attempts to demonize this form of labour it is becoming clear that its desirability as a form of employment is rising not falling. As one of the surrogates we interviewed lamented, many of those from lower castes are now having to compete for such work with those of higher social standing:

When I had done it at that time there was nothing, there were very few who would be doing it. But now all are doing it even the rich people are doing it. People from good families are also doing it. At first it was that people who are poor and do not have anything, those would do it, but now people, those who have everything, do it. (Shruti – egg donor and surrogate)

How and in what ways do these developments complicate existing accounts that position surrogates as a class of subaltern reproductive workers labouring to realize the desires of white, privileged parents who commission their work from the metropolitan centres of the West?

It is clear that regardless of whether they are located in India or the West, the vast majority of individuals who are commissioning surrogates are doing so as they are biologically incapable of producing their own children. It is often asserted that those who are infertile but wish to start families should instead adopt children. This is a line of argument with which I personally concur, in part, as such practices actively subvert the now canonical valorization of the genetic child. As the parent of two children to whom I have no biological relation I welcome the disruption of such overbearing norms. However, as much as I might wish it were not so, the desirability of the biologically related child remains a potent trope, one that is increasingly driving adoption – sadly not of children – but of ART. Indeed, it is this dynamic that compels women and men to pay the very substantial costs of infertility treatment (including the cost of supplied gametes) to produce a child to whom they remain at least partially genetically or biologically related. If the work of a ban on surrogacy is to end the purported indignity of paying others to sell their reproductive capacity, then surely that principle (and the associated ban) should be applied both commensurately and universally. This would result in the prohibition of all compensated exchanges of gametes for the purposes of assisted reproduction. I wonder how every parent of a donor-assisted IVF baby would respond to that prospect?

Finally, and perhaps most importantly of all, what of the surrogates’ motivations? To assert that such women are routinely exploited is also to presume that they exercise no agency in the decision making. To bear a child for another is no small undertaking. Whilst securing a substantial payment is a key inducement, it alone cannot be the only motivating factor. That is far too simplistic and unnuanced a reading. As Rudrappa notes (2012; Rudrappa & Forest, 2014), and as our own findings confirm, whilst many young women are invited or encouraged to become surrogates, most decline the offer, *despite* experiencing economic hardship. Clinics are, nevertheless, oversubscribed with potential applicants. As one clinical director explained to me, they turn down 14 applicants for every one they accept. Clinics have neither the need nor the desire to accept women for whom this would be a psychologically untenable journey. In this respect the surrogates are what I would describe as a ‘self-selecting’ cohort. They are typically those women (relatively small in number) who believe they can come to a physical and psychological accommodation with this form of labour.

The question of why they elect to undertake this work is central to analyses of the exploitation thesis. Such women often agree to become surrogates (in both India and the West) as they derive more personal meaning from this employment than they do from other, more oppressive, menial or generally inconsequential forms of work. Indeed, some women I would argue are motivated to perform this work as they view it as a form of *philanthropic labour*. For whilst commissioning parents may be well off financially, when it comes to fecundity they are most definitely ‘resource-poor’. Surrogates are women who must already have borne at least one, if not two, children of their own. Many have personally witnessed the social cost of infertility and the profound misery and social stigma experienced by those who are unable to produce a biological child. None would argue, as Ashenden (2013, p. 199) puts it, that ‘the product of a pregnancy can be easily alienated without remainder’; however, they are able to rationalize it, *in this context*, as an act of benefaction*.* As Bharti, a former surrogate and now agent^5^ explains:

The mother-feeling is felt in every [surrogate] woman; but it is a happy feeling that you are giving a child to someone who does not have her own child. An infertile woman suffers a lot of agony and pain. To give her a child is a very noble thing. (Bharti – agent, former surrogate)

One of the powerful implications of perpetuating racialized and gendered accounts of surrogacy that characterize the practitioners (the surrogates) as an oppressed and exploited minority is that they actively prohibit such women from occupying the role of benefactor of reproductive labour to the more privileged Indian or white Western women and men who avail themselves of their services. Keeping them in this role, whilst simultaneously denying the significance of their labour, works to strip them further of both power and self-respect.

How then could we more productively re-conceptualize the relations implicit in commercial surrogacy in India today? Ruth Sample argues (2003, p. 7) that we exploit others when we ‘make use of their genuine need for the sake of advantage in ways that fail to respect them’. In my view, it is when individuals (be they clinicians, commissioning parents or family members) actively collude to deliberately obscure or efface the vital work that the surrogate performs in realizing these important new forms of family building that genuine exploitation occurs. All too frequently the role of the surrogate and certainly the egg donor is completely ‘black-boxed’. The argument that foregrounding her role will disrupt the dynamics of nuclear family formation is, I think, both misplaced and dangerous. In fact, most of the psychosocial damage that arises from the practice of surrogacy – for commissioning parents, surrogates and surrogate children – arises, I would argue, from suppressing or denying the complex, entangled nature of the conception.

Assisted reproduction must, by its very nature, usher in new kinds of families, the very existence of which rely on, and generate, what I would call forms of ‘*distributed kinship*’. Rather than seeking to disavow these we must collectively learn to embrace them in ways that promote transparency and equity for all participants. In contemplating how we might more effectively undertake this work, we can profitably invoke some of the idioms and practices of intellectual property rights law. The fiction that the children of such conceptions are the inventions of a single romantic partnership must be put to the sword – as Rosemary Coombe (1996) suggests, new ‘authorial cartographies’ must be brought into play. I would argue that these children enter the world as what we might think of as ‘collaboratively authored works’, to whom all contributors should be accorded some degree of ‘open access’. Seeking to deny or obscure the investments that surrogates and egg donors make to the creation of the child is a form of exceptionalism that cannot be justly sustained.

## Conclusion

Much interest has been concentrated on surrogacy as a form of exceptionally exploitative reproductive labour that ought, by some accounts, to be banned outright. However, I hope, as I have shown here, the realities of lived experience that exist beyond the hyperbole of tabloid representations and their anxious protestations work to complicate such assertions. Laissez-faire approaches to regulation that some argue typify neoliberal modes of economic organization (but which I say are a common feature of all capitalist enterprises) generate forms of labour exploitation to be sure, but these are not, I argue, exceptional to clinical or reproductive labour practices. The argument for singling surrogacy out as an activity that demands exceptional forms of regulation (such as prohibition) is not, in my view, yet successfully sustained by appeal to empirical evidence.

Must commercial surrogacy always be exploitative of an underclass of women? As other studies have shown, and as our own also confirms, many of these women make deliberate and considered decisions to participate. Some do so under conditions of structural violence that constrain their life and employment choices, but these are amenable to reform through the implementation of strategies that will enhance rather than diminish the personal and political agency of such women. Those who seek to remove this form of employment must face the realities and accept the moral and political responsibility for the alternatives that these women will be forced to accept. For many this will involve labour in even more poorly regulated forms of work, all of which are less economically rewarding and potentially even more precarious and hazardous than surrogacy. In fact we know that surrogate workers themselves have been protesting actively against the proposed ban on commercial surrogacy in India since it was announced, on the basis that they will be stripped of one of the most lucrative revenue-generating opportunities they have or will ever encounter in their lifetimes.^6^ For these women, bans just invoke another form of ‘ethical paternalism’.

Where does this leave us then in terms of regulation? Options certainly exist to improve clinical practices within such clinics. Women have successfully lobbied in some countries for regulation of sex work as labour, and secured rights not only to sufficient wages but also medical care, occupational health and safety, work security and rights to set limits on the demands made by clients (Kempadoo & Doezema, 1998; Kotiswaran, 2008; Moukalif, 2009). Aligning surrogacy as a commensurate form of labour could provide the leverage to sustain similar regularization of this form of embodied labour. In some of the larger ART clinics in India these rights are already being secured. The women who undergo surrogacy there have already exercised their autonomy and their agency in arriving at a considered and informed judgement that their conditions of work, remuneration and care will be much better in the surrogacy clinic/workplace than they would be working in any of the other desperately menial and exploitative agricultural or industrial jobs that are their only other alternative forms of employment.

Constructing commercial surrogacy as an abject highly exploitative practice is, in my view, immensely problematic, and lumping it together uncritically with other equally poorly conceptualized practices such as ‘trafficking’ and ‘slavery’ does more harm than good. Certainly, the notion that a ban on commercial surrogacy will extinguish the practice is fanciful to say the least. Addressing the inequities in practice that we see in different corners of the globe requires thoughtfully constructed, nuanced and most importantly grounded regulation, not bumptious self-congratulatory bans that will only serve to worsen experiences and outcomes for those already undertaking this work. And after all, what could such a ban possibly achieve? India is now part of a highly interconnected and interdependent globalized fertility industry. If the tide of surrogacy goes out in India it will only wash up somewhere else, displacing the inequities that exist in one country onto unsuspecting others in alternative locations: Mexico, Cambodia, Nepal to name but a few.

Within and amongst all this, it is the surrogate, as Ashenden (2013, p. 212) astutely notes, ‘who is made to bear the burden of responsibility for society's ambivalence towards new reproductive practices’. Imagining into existence new ways of acknowledging the value of her work whilst simultaneously enhancing her legal and social protections remains a key objective, one that will require the application of supple conceptualizations of the moral and ethical dynamics at work in such practices rather than a retreat into the safe harbour of ‘exceptionalism’.

## Notes

1 As reported in *The Times of India* 15 October 2015. Available at: https://timesofindia.indiatimes.com/india/SC-suggests-ban-on-commercial-surrogacy/articleshow/49365734.cms.

2 In this respect I concur with arguments offered in Wilkinson (2016), although this work pre-dates that publication.

3 This research was undertaken under Wellcome Trust grant (097733/B/11/Z): *Investigating Reproductive Ethics-in-Context: The Indian Experience.* A total of 123 interviews with a range of key stakeholders including clinicians, hospital executives, private fertility centre managers, egg and sperm donors, surrogates, agents, commissioning parents, IVF consumers, pharmaceutical company representatives, policy makers and NGO advocates were completed between 2013 and 2017 in Jaipur, Mumbai, Delhi and Bangalore. Full ethical approval was obtained from King's College London (SSHL/12/13-26) and participating hospitals in India.

4 It is extremely interesting to note that almost all of the reports that offer negative accounts of the surrogate experience purport to focus on the pain of relinquishment for some, but not all surrogates. I would strongly argue, however, that it is not relinquishment *per se* that invokes such distress but rather the fact that the surrogate is refused access to any further information about the fate of their child; their ongoing health and welfare; lived experiences, etc. The politics of black-boxing the surrogate's relation to the child is taken up in a following section.

5 Agents are individuals (often but not always former surrogates) who recruit potential surrogates, represent their interests in negotiations on terms and conditions and who also steward them through the process of attending clinical appointments, etc. They typically receive a commission from the clinic for undertaking this work. The Indian Council of Medical Research Guidelines (2005) suggest that this work should be illegal; however, clinics thoroughly rely on the services of agents as they cannot by law recruit surrogates directly themselves.

6 See for example: https://www.theguardian.com/world/2016/jan/03/india-surrogate-embryo-ban-hardship-gujarat-fertility-clinic.
